# Auswirkungen des Cannabisgesetzes auf Bezugswege, Einstellungen und Wahrnehmungen von Konsumierenden

**DOI:** 10.1007/s00103-026-04258-y

**Published:** 2026-06-11

**Authors:** Larissa Steimle, Bernd Werse, Anke Stallwitz

**Affiliations:** 1 Fakultät Sozialwissenschaften, Technische Hochschule Nürnberg Georg Simon Ohm, Nürnberg, Bayern Deutschland; 2 Fachbereich Soziale Arbeit und Gesundheit, Frankfurt University of Applied Sciences, Frankfurt, Hessen Deutschland; 3 Evangelische Hochschule Freiburg, Freiburg, Baden-Württemberg Deutschland

**Keywords:** Cannabis, Drogenpolitik, Apotheke, Eigenanbau, Illegaler Markt, Cannabis, Drug policy, Pharmacy, Home cultivation, Illegal market

## Abstract

**Hintergrund:**

Mit dem Cannabisgesetz (CanG) verfolgte die Bundesregierung das Ziel, den Gesundheitsschutz zu verbessern und den illegalen Markt zurückzudrängen. Ein Teilerfolg des CanG würde sich daher in der Verschiebung von illegalen zu legalen Bezugswegen zeigen. Folgende Fragen standen im Zentrum: Wie haben sich die Bezugswege durch das CanG insgesamt und in verschiedenen Gruppen verändert? Welche Gruppen profitieren subjektiv am meisten durch das CanG?

**Methoden:**

Um die Forschungsfragen zu beantworten, wurde vom 24.03.2025 bis zum 10.06.2025 eine nichtrepräsentative, quantitative Online-Befragung mit Konsumierenden ab 14 Jahren durchgeführt. Die Teilnehmenden wurden zu ihren Bezugswegen vor und nach dem Inkrafttreten des CanG sowie zu den von ihnen wahrgenommenen Auswirkungen befragt. Die Stichprobe umfasste *N* = 11.471 Personen.

**Ergebnisse:**

Seit Inkrafttreten des CanG zeigte sich bei Erwachsenen insgesamt, vor allem aber bei Männern und Personen im ländlichen Raum, eine Verschiebung von illegal zu legal produziertem Cannabis, da Eigenanbau und Apothekenbezug stark zugenommen haben. Bei Jugendlichen zeigten sich kaum Veränderungen. Subjektiv wurden überwiegend positive Effekte des Gesetzes berichtet, wobei sich Geschlechter- und Regionalunterschiede zeigten, indem Männer sowie Personen aus Großstädten – insbesondere Berlin und Hamburg – häufiger positive subjektive Effekte wahrnahmen.

**Diskussion:**

Die Ergebnisse deuten darauf hin, dass für bestimmte Subgruppen Barrieren im Zugang zum legalen Markt bestehen, darunter Jugendliche, Frauen und die urbane Bevölkerung. Für diese Gruppen könnten gezielte Maßnahmen zum Abbau solcher Barrieren einen Beitrag zur weiteren Zurückdrängung illegaler Bezugswege leisten.

**Zusatzmaterial online:**

Zusätzliche Informationen sind in der Online-Version dieses Artikels (10.1007/s00103-026-04258-y) enthalten.

## Abstract (en)

**Background:**

With the Cannabis Act (CanG), the German government aimed to improve health protection and to curb the illegal cannabis market. A partial success of the CanG would be evident in the shift from illegal to legal supply channels. The following questions were central: How did sources of cannabis supply change overall and across different groups due to the Cannabis Act? Which groups subjectively benefit the most from the Cannabis Act?

**Methods:**

To address these research questions, a non-representative, quantitative online survey was conducted from 24 March 2025 to 10 June 2025 among cannabis users aged 14 and older. Participants were asked about their sources of supply before and after the Cannabis Act took effect as well as the subjectively perceived effects. The sample included *N* = 11,471 participants.

**Results:**

Since the CanG came into effect, a shift from illegally to legally produced cannabis has been observed among adults overall, particularly among men and people in rural areas, as home cultivation and pharmacy purchases have increased significantly. In contrast, little change was observed among adolescents. Participants reported predominantly positive subjective effects of the law. Gender and regional differences showed that men and residents of major cities—particularly Berlin and Hamburg—more frequently perceived positive subjective effects.

**Discussion:**

The results suggest that certain subgroups face barriers in accessing the legal market, including adolescents, women, and the urban population. For these groups, targeted measures aimed at reducing such barriers could contribute to further curbing illegal sources of supply.

## Hintergrund

Mit dem Cannabisgesetz (CanG) zielte die Bundesregierung unter anderem darauf ab, zu einem verbesserten Gesundheitsschutz beizutragen, den illegalen Markt für Cannabis einzudämmen und Jugendliche besser zu schützen (BT-Drs. 20/8704 S. 68). Mit dem Inkrafttreten zum 01.04.2024 hat sich nicht nur in Bezug auf den Besitz von Cannabis – für Erwachsene bis zu bestimmten Grenzen nun legal (§ 3 Konsumcannabisgesetz (KCanG)) –, sondern auch im Hinblick auf die Bezugswege einiges verändert. Durch das CanG dürfen Erwachsene bis zu 3 Cannabispflanzen pro Person anbauen (§ 9 KCanG). Zudem ist der gemeinschaftliche Anbau in sogenannten Anbauvereinigungen gestattet. Weiterhin wurde das Medizinal-Cannabisgesetze (MedCanG) (als Teil des CanG) geändert, sodass medizinisches Cannabis über eine gewöhnliche Verschreibung erhältlich ist, anstatt über ein Betäubungsmittelrezept verordnet werden zu müssen (§ 3 MedCanG).

Der Erfolg des CanG kann anhand von 2 Kriterien bewertet werden: durch Veränderungen der Bezugswege weg von illegalen (z. B. Dealer*innen) hin zu legalen Quellen (z. B. Eigenanbau) sowie durch eine positive Veränderung des subjektiven Empfindens von Konsumierenden.

In Bezug auf die erste Dimension (Änderung der Bezugswege hin zu legalen Quellen) lagen zum Zeitpunkt der Studie national keine Daten vor. Internationale Studien aus Regionen mit kommerziellem Verkauf zeigen, dass in US-Bundesstaaten Konsumierende überwiegend legale Angebote nutzten [1]. In Kanada stieg der Anteil legaler Bezugsquellen von unter 50 % im ersten Jahr (2019) auf 74 % im Jahr 2024, während nur 3–5 % primär illegale Quellen nutzten [2]. In Uruguay wurde in Studien ein deutlicher Anstieg des Eigenanbaus berichtet [3], begleitet von einem „grauen Markt“ aus legal produziertem, aber illegal weiterverkauftem Cannabis [4]. Umsatzschätzungen sahen Cannabis Social Clubs als wichtigste Quelle [5]. Um die Auswirkungen des CanG auf die Bezugswege in Deutschland besser zu verstehen, sollte die Studie die folgende Frage beantworten: *Wie haben sich Bezugswege durch das CanG insgesamt und in verschiedenen Gruppen verändert?*

Ein weiterer Erfolgsindikator für das CanG ist die Frage nach subjektiven Auswirkungen auf Konsumierende. Im internationalen Raum gibt es hierzu nur wenige direkte Forschungsergebnisse. Am nächsten kommen dieser Frage Studien zu Effekten auf die psychische Gesundheit. Eine internationale Metastudie kam hier zu uneindeutigen Ergebnissen [6], während eine weitere Studie über negative Effekte bei Jüngeren und positive Effekte bei älteren Erwachsenen berichtete [7]. Zudem deuten internationale wie auch deutsche Studien darauf hin, dass Stigmatisierung von Konsumierenden erhebliche Belastungen und Probleme verursachen kann [8, 9]. Auch die tendenziell höhere Neigung von Cannabiskonsumierenden zu paranoiden Gedanken [10] könnte durch die Kriminalisierung verstärkt worden sein. An verschiedenen Stellen wurde die Hoffnung formuliert, dass Konsumierende durch das CanG weniger Stigmatisierung erfahren, strafverfolgungsbezogene Belastungen reduziert werden und der Zugang zu Unterstützungsangeboten leichter fällt [11]. Um dies näher zu eruieren, beschäftigte sich die vorliegende Studie auch mit der Frage: *Welche Gruppen profitieren subjektiv am meisten durch das CanG?*

## Methode

Zur Beantwortung der Forschungsfragen wurde vom 24.03. bis 10.06.2025 eine quantitative, nichtrepräsentative Online-Befragung mit dem Titel „Veränderungen für Konsumierende von Cannabis durch das Cannabisgesetz“ (KonCanG) durchgeführt. Teilnehmen konnten Personen ab 14 Jahren, die seit Inkrafttreten des CanG (01.04.2024) Tetrahydrocannabinol-(THC-)haltiges Cannabis konsumiert hatten. Ausgeschlossen waren Konsumierende ausschließlich (teil-)synthetischer Cannabinoide. Die Befragung wurde von der Frankfurt University of Applied Sciences und der Evangelischen Hochschule Freiburg durchgeführt.

Die Rekrutierung erfolgte über Social Media (vor allem X, aber auch z. B. Bluesky), Fachorganisationen (z. B. Landesstellen für Suchtfragen, Dachorganisationen der Cannabis Social Clubs), Pressemitteilungen und Newsletter. Die verschiedenen Organisationen und Akteur*innen wurden von den Autor*innen kontaktiert und um eine Verbreitung der Studie gebeten. Zudem wurden gezielt Akteur*innen aus den Bereichen Drogenpolitik und Cannabisaktivismus angesprochen. Ziel war eine große, jedoch nicht repräsentative Stichprobe.

Der Fragebogen enthielt maximal 41 Fragen, wobei die Anzahl der Fragen in Abhängigkeit der gegebenen Antworten variierte. Jugendliche und Erwachsene erhielten aufgrund unterschiedlicher rechtlicher Rahmenbedingungen und typischer Bezugswege separate Frageblöcke. Der Fragebogen steht als Onlinematerial zur Verfügung. Jugendspezifische Fragen sind an der Angabe der Altersbedingung „älter als 14, jünger als 18 Jahre“ erkennbar. Die Befragung dauerte ca. 10 min. Für die Durchführung der Befragung wurde die Software Questionstar genutzt.

Die Datenauswertung erfolgte mit SPSS (Version 22, IBM, Armonk, NY, USA). Die Auswertungen folgten einem deskriptiv-explorativen Ansatz. Diesem Ansatz entsprechend lag der Fokus der Auswertung – neben einer Angabe der Häufigkeiten – vor allem auf dem Testen von Unterschieden in den einzelnen Subgruppen. Dabei wurde jeweils auf folgende Unterschiede getestet: Alter (Jugendliche/Erwachsene), Geschlecht, Ortsgröße und Bundesland. Insgesamt orientierte sich die Auswertung an 2 Zielkriterien: *(1) Veränderung der Bezugswege insgesamt und in verschiedenen Gruppen* sowie (2) *subjektive Auswirkungen auf unterschiedliche Gruppen*.

*Zu (1): *Im Hinblick auf die Bezugswege wurden die Befragten unter anderem zu ihrer wichtigsten Hauptquelle vor (Q22, Tab. Z1) und nach (Q29, Tab. Z1) Inkrafttreten des CanG befragt. Für die Analyse werden zunächst Häufigkeiten für beide Zeitpunkte berichtet und anschließend die genannten Subgruppenvergleiche (Alter, Geschlecht, Ortsgröße und Bundesland) vorgenommen (jeweils mit Chi-Quadrat-Tests und den zugehörigen Effektgrößen (Cramers V)). Jugendliche und Erwachsene erhielten getrennte Frageblöcke in Bezug auf Hauptbezugswege, weshalb ein direkter statistischer Vergleich nicht möglich ist. Die Ergebnisse werden daher deskriptiv gegenübergestellt. Die Veränderungen zwischen den beiden Zeitpunkten wurden mittels McNemar-Tests auf Basis desselben Personenstichprobenpaares (vorher vs. nachher) berechnet.

*Zu (2):* In Bezug auf die subjektiven Auswirkungen des CanG wurden den Befragten 5 Aussagen zur Bewertung auf einer 5‑stufigen Likert-Skala vorgelegt (*stimme überhaupt nicht zu* bis *stimme voll und ganz zu*; Q33, s. Onlinematerial). Die Aussagen bezogen sich auf wahrgenommene Beobachtung beim Konsum, Akzeptanz, Angst vor Strafverfolgung, Bereitschaft zur Hilfesuche sowie Sorgen im Straßenverkehr. Für jede Aussage wurden Mittelwerte, Mediane und Streuungen berechnet und anschließend Subgruppenvergleiche vorgenommen. Berücksichtigt wurden Geschlecht, Ortsgröße und Bundesland. Aufgrund geringer Beteiligung Jugendlicher und fehlender Vergleichbarkeit aufgrund unterschiedlicher Fragen wurde auf einen Altersvergleich verzichtet. Mittelwertvergleiche erfolgten mittels einfaktorieller Varianzanalysen (ANOVA, inkl. Tukey-Post-hoc-Tests).

Das Signifikanzniveau wurde auf α = 0,05 festgelegt. Da es sich um eine explorative Studie handelt, wurden keine Adjustierungen für multiple Tests vorgenommen. Die erhöhte Wahrscheinlichkeit von Alpha-Fehlern („inflationierte familywise error rate“) ist zu berücksichtigen. Alle inferenzstatistischen Ergebnisse sind daher als Hinweise auf mögliche Zusammenhänge zu verstehen und sollten in zukünftigen Studien repliziert werden. Sowohl signifikante als auch nicht signifikante Ergebnisse werden berichtet.

## Ergebnisse

Der Fragebogen wurde 13.919-mal begonnen und 11.872-mal vollständig ausgefüllt. Davon wurden 401 Bögen ausgeschlossen (u. a. wegen fehlenden Konsums, ungültiger Altersangaben, fehlenden Geschlechts oder Wohnsitzes im Ausland), sodass 11.471 Fragebögen in die Auswertung eingingen. Im Folgenden beziehen sich die Verweise auf einzelne Fragen (Q) stets auf den Fragebogen im Onlinematerial.

### Stichprobenbeschreibung

85,9 % der Stichprobe (*N* = 11.471) waren männlich (Tab. 1), 13,4 % weiblich und 0,7 % divers (Q35). Männer waren damit überrepräsentiert, was zwar der stärkeren Präsenz von Männern unter intensiv Konsumierenden entspricht, allerdings nicht in diesem Ausmaß [12, 13].

**Tab. 1:** Stichprobe – Basisdaten

	Werte (absoluter Anteil)	Gesamtzahl ausgewerteter Fragebögen
Männliches Geschlecht	85,9 % (*n* = 9851)	11.471
Durchschnittsalter Erwachsene	37 Jahre (*n* = 11.375)	11.471
Universitäts- oder Hochschulabschluss	31,9 % (*n* = 3658)	11.435
Haushaltsnettoeinkommen über 3000 €	49,3 % (*n* = 5625)	11.404
Deutsche Staatsbürgerschaft	96,5 % (*n* = 11.039)	11.442

0,8 % der Befragten waren Jugendliche (Mittelwert (M) = 16,5 Jahre; Standardabweichung (SD) = 0,74; Median (Me) = 17 Jahre), 99,2 % Erwachsene (M = 37; SD = 10,64; Me = 37, Q6). Das Durchschnittsalter lag unter dem Bevölkerungsdurchschnitt (44,9 Jahre; [14]), aber höher als bei regelmäßig bis häufig Konsumierenden [12, 13].

Von den Befragten (*n* = 11.452) hatten 31,3 % eine Berufsausbildung, 31,9 % einen Hochschulabschluss und 19,5 % Abitur/Fachhochschulreife (Q36). 10,8 % besaßen einen Realschulabschluss, 3,9 % einen Hauptschulabschluss, 0,9 % waren noch in der Schule, 0,5 % hatten keinen Abschluss. 1,3 % machten Freitextangaben, meist zu Weiterqualifizierungen. Das Bildungsniveau der Stichprobe lag über dem der Gesamtbevölkerung [15].

Beim monatlichen Nettoeinkommen (*n* = 11.404, Q37) gaben 16,6 % 0–1500 €, 34 % 1501–3000 €, 31,9 % 3001–5000 € und 17,4 % 5001 € oder mehr an. Im Vergleich ergibt sich ein ähnlicher Median wie in der Gesamtbevölkerung (3200 € [16]).

96,5 % der Befragten (*n* = 11.442) hatten die deutsche Staatsbürgerschaft, 1,9 % eine doppelte und 1,6 % eine andere Staatsbürgerschaft (Q38). Personen ohne deutsche Staatsbürgerschaft (ca. 15 % in der Gesamtbevölkerung [17]) waren deutlich unterrepräsentiert.

37,1 % der Befragten (*n* = 11.443) lebten in einer Großstadt (> 100.000 Einwohner*innen; Gesamtbevölkerung: 32,03 % [18]), 31,1 % in mittelgroßen Städten (10.000–100.000 Einwohner*innen; GB: 42,77 % [18]) und 31,8 % kleinen Städten/Dörfern (< 10.000 Einwohner*innen; GB: 25,2 % [18]; Q39). Damit waren kleine und große Gemeinden leicht überrepräsentiert, mittlere unterrepräsentiert.

21,9 % der Gesamtstichprobe (*N* = 11.471) lebten in Bayern, 19,4 % in Nordrhein-Westfalen, 14,2 % in Baden-Württemberg, 9,3 % in Niedersachsen, 8,8 % in Hessen, 5,3 % in Rheinland-Pfalz, 4,4 % in Berlin, 3,3 % in Sachsen, 2,8 % in Schleswig-Holstein, 2,6 % in Hamburg, 1,6 % in Brandenburg, 1,6 % in Thüringen, 1,5 % in Sachsen-Anhalt, 1,2 % in Mecklenburg-Vorpommern, 1,2 % im Saarland und 0,8 % in Bremen (Q40). Ostdeutsche Länder waren insgesamt unterrepräsentiert, während Bayern im Vergleich zur Gesamtbevölkerung überdurchschnittlich stark vertreten war [19].

Von der Gesamtstichprobe (*N* = 11.471) hatten bereits 97,5 % der Befragten vor dem 01.04.2024 Cannabis konsumiert, während 2,5 % nach dem Inkrafttreten des Gesetzes erstmals konsumierten (Q4).

39,2 % der Befragten (*N* = 11.471) konsumierten zum Zeitpunkt der Befragung täglich, 28,8 % mehrmals pro Woche (aber nicht täglich), 12,8 % ungefähr einmal pro Woche, 11,8 % nicht wöchentlich, aber mindestens einmal pro Monat und 7,3 % nicht monatlich, aber mindestens einmal pro Jahr (Q8). Im Mittel wurden von denjenigen, die angaben, täglich Cannabis zu konsumieren (*n* = 4403), 1,26 g pro Tag konsumiert (Q9).

### Hauptbezugswege vor Inkrafttreten des Cannabisgesetzes

Gefragt nach der Hauptquelle, aus der Erwachsene (*n* = 11.262) vor dem 01.04.2024 ihr Cannabis bezogen hatten (Q22), gaben 37,2 % bekannte/vertraute Dealer*innen an (Tab. 2). Auf Platz 2 lagen mit 26,2 % Freund*innen/Bekannte, die Cannabis nicht selbst angebaut hatten, gefolgt von Eigenanbau mit 9,4 %, Freund*innen/Bekannte, die Cannabis selbst angebaut hatten, mit 8,9 %, Apotheken mit 5,2 %, Dealer*innen in der Öffentlichkeit mit 4,4 %, Ausland mit 2,6 %, Darknet mit 1,9 % und Social Media mit 0,7 %. 0,8 % gaben sonstige Quellen an. 2,6 % gaben an, dass sie zu dieser Zeit nicht konsumiert hatten.

**Tab. 2:** Hauptbezugswege vor Inkrafttreten des Cannabisgesetzes am 01.04.2024 nach Geschlecht (Erwachsene)

Hauptquelle vor dem 01.04.2024	Insgesamt (*n* = 11.262) (%)	Männlich (*n* = 9683; %)	Weiblich (*n* = 1499; %)	Divers (*n* = 80; %)
Bekannte/vertraute Dealer*innen	37,2	38,1	32,0	27,5
Freund*innen/Bekannte (KEIN Eigenanbau)	26,2	24,9	33,8	33,8
Eigenanbau	9,4	10,0	5,3	11,3
Freund*innen/Bekannte (Eigenanbau)	8,9	8,6	10,9	10,0
Apotheke	5,2	5,3	4,9	5,0
Dealer*innen in der Öffentlichkeit	4,4	4,5	3,9	1,3
Kein Konsum	2,6	2,4	3,7	5,0
Ausland	2,6	2,5	2,9	3,8
Darknet/Internet	1,9	2,1	0,7	1,3
Social Media	0,7	0,8	0,6	1,3
Sonstiges	0,8	0,8	1,1	0,0

Gefragt nach der Hauptquelle, aus der Jugendliche (*n* = 96) vor dem 01.04.2024 ihr Cannabis bezogen hatten (Q17), gaben 14,6 % der Befragten an, zu dieser Zeit nicht konsumiert zu haben. Ansonsten bezogen die meisten Jugendlichen ihr Cannabis mit 41,7 % von Freund*innen/Bekannten, gefolgt von 32,3 %, die ihr Cannabis von bekannten und vertrauten Dealer*innen bezogen. 9,4 % nannten weitere Bezugswege.

In Bezug auf die Hauptbezugsquellen der Erwachsenen vor dem 01.04.2024 zeigten sich Geschlechterunterschiede, wobei sich mehrere signifikante, jedoch durchweg kleine Effekte zeigten: Frauen berichteten im Vergleich zu Männern häufiger den Bezug über Freund*innen/Bekannte ohne Eigenanbau (χ^2^(1) = 53,19, *p* < 0,001, V = 0,069) und mit Eigenanbau (χ^2^(1) = 8,42, *p* = 0,004, V = 0,027), während Männer häufiger bekannte Dealer*innen (χ^2^(1) = 20,63, *p* < 0,001, V = 0,043), Internetquellen (χ^2^(1) = 13,39, *p* < 0,001, V = 0,035) und Eigenanbau (χ^2^(1) = 32,71, *p* < 0,001, V = 0,054) nutzten. Für andere Bezugsquellen ergaben sich keine signifikanten Geschlechterunterschiede, darunter unbekannte Dealer*innen (χ^2^(1) = 1,09, *p* = 0,30, V = 0,010), Social Media (χ^2^(1) = 0,47, *p* = 0,492, V = 0,007), Apotheken (χ^2^(1) = 0,30, *p* = 0,58, V = 0,005) und Ausland (χ^2^(1) = 1,04, *p* = 0,31, V = 0,010). Die Verteilungen innerhalb der Kategorie Divers ähnelten teilweise denjenigen von Männern, sind aufgrund geringer Fallzahlen jedoch nur eingeschränkt interpretierbar. Insgesamt lagen alle Effektstärken im kleinen Bereich (Cramers V ≤ 0,074), sodass die beobachteten Unterschiede trotz statistischer Signifikanz inhaltlich als gering einzustufen sind.

Zwischen der Ortsgröße und den vor dem 01.04.2024 genutzten Hauptbezugsquellen zeigten paarweise Vergleiche jeweils signifikante, jedoch durchweg sehr kleine Unterschiede (Dorf vs. Mittelstadt: χ^2^(10) = 41,95, *p* < 0,001, V = 0,077; Mittelstadt vs. Großstadt: χ^2^(10) = 92,55, *p* < 0,001, V = 0,109; Dorf vs. Großstadt: χ^2^(10) = 29,25, *p* = 0,001, V = 0,061). Es wurden keine direkten statistischen Vergleiche vorgenommen. Inhaltlich zeigte sich auf deskriptiver Ebene, dass Personen aus Dörfern etwas häufiger Eigenanbau und den Bezug über Freund*innen mit Eigenanbau nutzten, während Großstädter*innen häufiger bekannte Dealer*innen sowie – in geringem Maße – Social Media als Bezugsquelle angaben.

Zwischen den Bundesländern zeigten sich signifikante Unterschiede in den vor dem 01.04.2024 genutzten Hauptbezugsquellen (χ^2^(150) = 742,996, *p* < 0,001, V = 0,081). Die Effektstärke war jedoch klein, sodass die Unterschiede inhaltlich gering ausfielen. Es wurden keine direkten statistischen Vergleiche vorgenommen. Deskriptiv fanden sich lediglich leichte Muster, zum Beispiel etwas häufiger Dealer*innen in Stadtstaaten oder leicht erhöhte Eigenanbauanteile in einigen ostdeutschen Flächenländern.

### Hauptbezugswege nach Inkrafttreten des Cannabisgesetzes

In Bezug auf die Hauptquelle, aus der die Erwachsenen (*n* = 11.375) in den letzten 6 Monaten ihr Cannabis bezogen hatten (Q29), gaben 49 % Eigenanbau als Hauptquelle an, gefolgt von Apotheken mit 29,2 % (Tab. 3). Danach folgten Freund*innen/Bekannte, die selbst Cannabis angebaut hatten, mit 8,3 %, Freund*innen/Bekannte, die Cannabis nicht selbst angebaut hatten, mit 4,7 %, bekannte/vertraute Dealer*innen mit 4,7 %, Anbauvereinigungen mit 1,9 %, Ausland mit 0,6 %, Dealer*innen in der Öffentlichkeit mit 0,3 %, Darknet/Internet mit 0,3 % und Social Media mit 0,2 %. 0,8 % wählten die Kategorie „Sonstiges“.

**Tab. 3:** Hauptbezugswege in den letzten 6 Monaten nach Geschlecht (Erwachsene)

Hauptbezugswege in den letzten 6 Monaten	Insgesamt (*n* = 11.375; %)	Männlich (*n* = 9782; %)	Weiblich (*n* = 1511; %)	Divers (*n* = 82; %)
Eigenanbau	49,0	51,5	33,5	37,8
Apotheke	29,2	29,9	25,0	29,3
Freund*innen/Bekannte (Eigenanbau)	8,3	7,0	16,5	9,8
Freund*innen/Bekannte (KEIN Eigenanbau)	4,7	3,5	11,9	11,0
Bekannte/vertraute Dealer*innen	4,7	4,2	8,1	7,3
Anbauvereinigung	1,9	1,9	1,7	2,4
Ausland	0,6	0,5	1,0	1,2
Dealer*innen in der Öffentlichkeit	0,3	0,3	0,6	1,2
Darknet/Internet	0,3	0,3	0,3	0,0
Social Media	0,2	0,2	0,2	0,0
Sonstiges	0,8	0,7	1,1	0,0

Gefragt nach der Hauptquelle, aus der Jugendliche (*n* = 96) aktuell ihr Cannabis beziehen (Q14), nannten 45,8 % der Befragten Freund*innen/Bekannte, 33,3 % vertraute und bekannte Dealer*innen, 6,3 % Eigenanbau und 5,2 % Dealer*innen in der Öffentlichkeit. 9,3 % nutzten weitere Quellen.

Im Hinblick auf die Hauptbezugswege der Erwachsenen in den letzten 6 Monaten zeigten sich signifikante, jedoch durchweg kleine Geschlechterunterschiede: Frauen berichteten im Vergleich zu Männern häufiger den Bezug über Freunde mit Eigenanbau (χ^2^(1) = 155,97; *p* < 0,001, *V* = 0,118), Freunde ohne Eigenanbau (χ^2^(1) = 208,52, *p* < 0,001, *V* = 0,136) sowie aus dem Ausland (*χ*^*2*^(1) = 5,95, *p* = 0,015, *V* = 0,023). Diverse Personen bezogen ebenso signifikant häufiger ihr Cannabis über Freund*innen, die nicht selbst anbauen, im Vergleich zu Männern (*χ*^*2*^(1) = 13,11, *p* < 0,001, *V* = 0,036). Auch der Bezug über bekannte Dealer*innen (χ^2^(1) = 46,02; *p* < 0,001, *V* = 0,064) sowie über Dealer*innen in der Öffentlichkeit (χ^2^(1) = 4,61; *p* = 0,032, V = 0,020) war unter Frauen häufiger. Männer bauten im Vergleich zu Frauen häufiger selbst an (*χ*^*2*^(1) = 170,73, *p* < 0,001, V = 0,123) und nutzen zudem häufiger Apotheken (*χ*^*2*^(1) = 15,10, *p* < 0,001, V = 0,037).

Zwischen der Ortsgröße und den aktuell genutzten Hauptbezugsquellen zeigte sich ein statistisch signifikanter, jedoch sehr kleiner Zusammenhang (Dorf vs. Mittelstadt: χ^2^(10) = 55,36, *p* < 0,001, V = 0,088; Mittelstadt vs. Großstadt: χ^2^(10) = 28,71, *p* < 0,001, V = 0,061; Dorf vs. Großstadt: χ^2^(10) = 146,29, *p* = 0,001, V = 0,137). Inhaltlich zeigte sich auf deskriptiver Ebene, dass Personen aus kleineren Gemeinden häufiger Eigenanbau als Hauptquelle angaben, während in mittelgroßen und besonders großen Städten der Bezug über Apotheken, Freund*innen/Bekannte sowie bekannte Dealer*innen häufiger vorkam.

Zwischen den Bundesländern und den aktuell genutzten Hauptbezugsquellen zeigte sich ein statistisch signifikanter, jedoch sehr kleiner Zusammenhang (*χ*^*2*^(150) = 573,83, *p* < 0,001; *V* = 0,071). Es wurden wiederum keine direkten paarweisen Vergleiche zwischen einzelnen Bundesländern vorgenommen, sodass die folgenden Unterschiede ausschließlich deskriptiv zu interpretieren sind: In mehreren Flächenländern (z. B. Baden-Württemberg, Bayern, Niedersachsen) wurde Eigenanbau vergleichsweise häufiger als Hauptquelle angegeben, während Stadtstaaten wie Berlin oder Hamburg etwas höhere Anteile bei bekannten Dealer*innen, Freunden/Bekannten sowie einzelnen seltenen Quellen aufwiesen. Digitale Beschaffungswege (Darknet, Social Media), Ausland und Anbauvereinigungen blieben in allen Bundesländern insgesamt selten.

### Vergleich der Hauptbezugswege vor und nach Inkrafttreten des Cannabisgesetzes

**Abb. 1:**
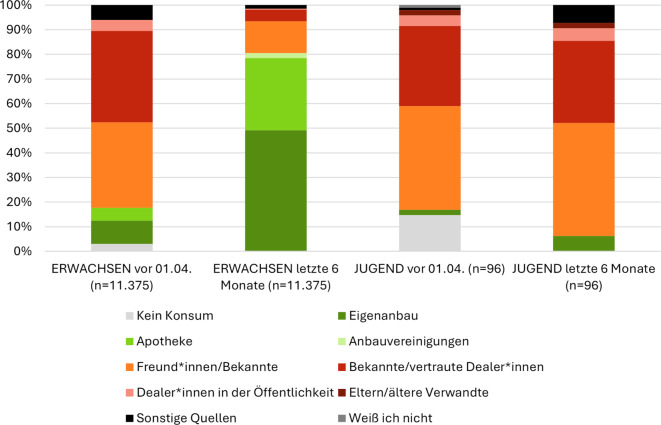
Vergleich der Hauptbezugswege von Cannabis bei Jugendlichen und Erwachsenen vor Inkrafttreten des Cannabisgesetzes am 01.04.2024 und in den letzten 6 Monaten. (Aus Gründen der Übersichtlichkeit wurden die Kategorien „Freund*innen mit Eigenanbau“ und „Freund*innen ohne Eigenanbau“ zu „Freund*innen/Bekannte“ zusammengefasst. Bei Erwachsenen entfiel die Option „Eltern/ältere Verwandte“, bei Jugendlichen die Option „Anbauvereinigungen“. Die Kategorien „Ausland“, „Darknet/Internet“ und „Social Media“ wurden aufgrund geringer Relevanz unter „Sonstige Quellen“ zusammengefasst)

Vergleicht man die Hauptbezugswege vor dem 01.04.2024 mit denen der letzten 6 Monate, zeigt sich ein Anstieg der Verwendung legaler Quellen (Abb. 1): Hatten vor dem CanG nur 23,5 % der Erwachsenen Cannabis hauptsächlich aus Quellen bezogen, die nunmehr (ursprünglich) legal sind, waren es seit dem Gesetz 88,4 %. Mit „nunmehr (ursprünglich) legal“ sind Quellen mit legalem Ursprung gemeint, also Eigenanbau, Apotheken, Freund*innen/Bekannte (Eigenanbau) und Anbauvereinigungen. Nach Inkrafttreten des CanG hat sich der Eigenanbau als Hauptbezugsquelle signifikant erhöht (McNemar-Test, *p* < 0,001): 4684 Personen gaben ihn neu als Hauptquelle an, während 172 ihn nicht mehr nannten. Auch der Apothekenbezug stieg als Hauptbezugsquelle signifikant an (McNemar-Test, *p* < 0,001; 2884 Neuangaben vs. 151 Abgänge). Der Bezug über Freund*innen, die selbst anbauten, blieb als Hauptquelle unverändert (McNemar-Test, nicht signifikant). Dagegen ging der Bezug über Freund*innen ohne Eigenanbau als genannte Hauptquelle signifikant zurück (McNemar-Test, *p* < 0,001). Die Zahl der Personen, die den Bezug von Cannabis von bekannten Dealer*innen als Hauptquelle angaben, sank signifikant (McNemar-Test, *p* < 0,001). Vergleicht man nun die Hauptbezugswege der Jugendlichen vor und nach dem Inkrafttreten des CanG, zeigen sich keine signifikanten Veränderungen (McNemar-Test, *p* > 0,05).

### Subjektive Veränderungen in Einstellungen und Empfinden bei Erwachsenen

**Abb. 2:**
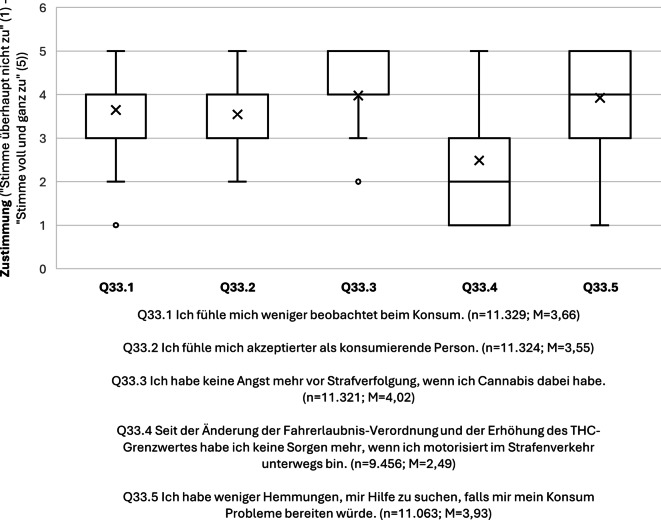
Veränderungen cannabisbezogener Wahrnehmungen/Empfindungen seit der Einführung des Cannabisgesetzes (Boxplots). Der Median ist mit X gekennzeichnet

Weiterhin wurden die Befragten nach den subjektiv empfundenen Auswirkungen des CanG gefragt (Q33). Die Ergebnisse sind in Abb. 2 dargestellt. Vor dem Hintergrund potenziell sozial erwünschten Antwortverhaltens bei subjektiven Veränderungen stehen vor allem die Unterschiede zwischen den Gruppen im Fokus.

Eine Analyse der Unterschiede zwischen den Bundesländern ergab, dass Konsumierende in Bayern und Nordrhein-Westfalen im Vergleich zu jenen in Niedersachsen etwas häufiger angaben, sich weniger beobachtet zu fühlen als zuvor, wobei der Unterschied zwar statistisch signifikant (Bayern vs. Niedersachsen *p* = 0,024; Nordrhein-Westfalen vs. Niedersachsen *p* < 0,001), aber mit einer sehr kleinen Effektgröße (*η*^2^ = 0,003) praktisch kaum relevant war. Darüber hinaus zeigte sich im Vergleich zu verschiedenen Bundesländern, dass sich Personen aus Berlin und Hamburg seit dem CanG signifikant sicherer vor Strafverfolgung fühlten (*p* < 0,001), was vermuten lässt, dass Ängste vor Strafverfolgung in Großstädten deutlicher abgenommen haben könnten (*η*^2^ = 0,006). Dies bestätigt sich mit Blick auf die Ortsgröße: Befragte in Großstädten stimmten deutlich eher dieser Aussage zu (M = 4,19; SD = 1,04) als Befragte in Mittelstädten (M = 3,98; SD = 1,16) und Kleinstädten bzw. Dörfern (M = 3,87; SD = 1,19; ANOVA F(2) = 83,84, *p* < 0,001). Weitere Unterschiede zeigten sich bei der Aussage, weniger Hemmungen zu haben, sich Hilfe zu suchen: Hier berichteten insbesondere Befragte aus Bayern im Vergleich zu verschiedenen Bundesländern signifikant seltener eine gestiegene Bereitschaft zum Hilfesuchen (*η*^2^ = 0,006).

Die einfaktoriellen ANOVAs zeigten bei allen Aussagen signifikante Geschlechterunterschiede (meist *p* < 0,001; Straßenverkehr: *p* = 0,002): Mit Ausnahme der Aussage über Cannabis im Straßenverkehr stimmen Männer sowie diverse Personen allen Aussagen im Schnitt etwas häufiger zu als Frauen.

## Diskussion

Die Stichprobe (*N* = 11.471) unterscheidet sich klar von der Gesamtbevölkerung: Sie ist jünger, häufiger männlich, besser gebildet, weniger migrantisch und stärker in Westdeutschland verortet. Alter und Geschlecht spiegeln zumindest in der Tendenz typische Merkmale Cannabiskonsumierender wider [12], auch wenn im Vergleich zu den Cannabiskonsumierenden im Epidemiologischen Suchtsurvey die Stichprobe stärker männlich geprägt und etwas älter ist [13].

Das höhere Bildungsniveau und die geringere migrantische Beteiligung dürften auf eine geringere Teilnahmebereitschaft [20] und die Rekrutierungswege zurückzuführen sein. Das Haushaltsnettoeinkommen ist ähnlich dem der Gesamtbevölkerung. Über zwei Drittel konsumieren täglich oder mehrmals wöchentlich. Da der Großteil des Gesamtbedarfs vermutlich auf diese Gruppe häufig Konsumierender zurückgeht, bieten die Daten gute Hinweise zur Änderung des gesamten deutschen Cannabismarktes [21]. Es wurden nur wenige Jugendliche erreicht (< 100), was auf die Rekrutierungswege, möglicherweise aber auch auf die geringe und weiter sinkende Popularität von Cannabis in dieser Altersgruppe zurückzuführen ist [22–24].

### Bezugswege

Seit Inkrafttreten des CanG zeigen sich bei Jugendlichen nur geringe Veränderungen im Hinblick auf die Bezugswege, während bei Erwachsenen eine deutliche Verlagerung zu legalen Bezugsquellen erkennbar ist. Vor dem Gesetz bezogen nur 23,5 % ihr Cannabis aus ursprünglich legalen Quellen (Eigenanbau, Apotheken, Freund*innen/Bekannte (Eigenanbau) und Anbauvereinigungen), zum Zeitpunkt der Befragung waren es 88,4 %. Besonders der Eigenanbau hat stark zugenommen: Fast die Hälfte der Befragten nutzt ihn als Hauptquelle (vor CanG 9,4 %). Auch Apotheken haben deutlich an Bedeutung gewonnen, was auf den erleichterten Zugang zu Privatrezepten zurückzuführen sein könnte. Im 1. Zwischenbericht zur Evaluation des Konsumcannabisgesetzes (EKOCAN), der Ende September 2025 veröffentlicht wurde, wird berichtet, dass Medizinalcannabis in den 12 Monaten nach Inkrafttreten von KCanG und MedCanG geschätzte 12–14 % des Gesamtbedarfs deckte [25]. Auch der 2. EKOCAN-Zwischenbericht (veröffentlicht Anfang April 2026) bestätigt die große Relevanz von Medizinalcannabis [26]. Der Bezug über Dealer*innen und Bekannte ist zurückgegangen – mit Ausnahme der Weitergabe von legal angebautem Cannabis durch Freund*innen. Dies deckt sich auch mit dem 1. EKOCAN-Zwischenbericht, wonach „social supply“ – gemeint ist die Weitergabe von Cannabis im sozialen Umfeld – eine zentrale Rolle einnimmt [25].

In der in diesem Artikel vorgestellten Befragung spielte der Bezug über Anbauvereinigungen kaum eine Rolle und auch laut dem 1. EKOCAN-Zwischenbericht produzierten die Anbauvereinigungen 2024 weniger als 0,1 % des konsumierten Cannabis und bis April 2025 waren höchstens 2 % der konsumierenden Erwachsenen dort Mitglied [25]. Im Jahr 2025 konnten 3,5 % der Konsumierenden ihr Cannabis aus einer Anbauvereinigung beziehen [26]. Fraglich bleibt, ob der Einfluss steigen wird, zumindest, solange Apotheken und Eigenanbau gut zugänglich bleiben. Während Anbauvereinigungen stark reguliert sind, bieten Apotheken eine breite Produktpalette, geringe Preise und sogar Lieferungen nach Hause, sodass Anbauvereinigungen unter Umständen kaum konkurrenzfähig sind.

Betrachtet man die einzelnen Gruppen genauer, zeigen sich Unterschiede, die allerdings durchweg klein ausfielen: Auf legale Bezugswege greifen vor allem Erwachsene sowie Personen, die in wenig urbanen Räumen leben, zurück. Weiterhin zeigen sich Geschlechterunterschiede. Männer dominieren sowohl beim Eigenanbau als auch bei der Nutzung von Apotheken. Frauen hingegen beziehen ihr Cannabis häufiger über Freund*innen und Bekannte, was mit der insgesamt geringeren Beteiligung an der aktiven Beschaffung zusammenhängen dürfte [27].

### Veränderungen in der subjektiven Wahrnehmung

Auch auf subjektiver Ebene zeigen sich Veränderungen. Interessant sind in diesem Zusammenhang die Gruppenvergleiche. Die Unterschiede zwischen den Bundesländern sind signifikant, aber mit extrem kleinen Effektgrößen. Berlin und Hamburg verzeichnen den stärksten Rückgang der Strafverfolgungsangst und Bayern die geringste Veränderung in der Bereitschaft, Hilfe zu suchen. Obwohl sich insbesondere in Großstädten eine eher hohe Nutzung illegaler Quellen zeigt, profitieren urbane Räume am stärksten im subjektiven Sicherheitserleben. Es zeigten sich außerdem Geschlechterunterschiede, indem Männer und diverse Personen im Vergleich zu Frauen subjektiv stärker profitieren.

### Limitationen

Die Erhebung ist nicht repräsentativ. Häufig konsumierende Personen, auf die ein Großteil des Cannabiskonsums entfällt [21], wurden jedoch gut erreicht, was die Aussagekraft zur Verschiebung von illegalen zu legalen Bezugsquellen stärkt. Der Rekrutierungsweg begünstigt eine Überrepräsentation drogenpolitisch interessierter Personen, was sozial erwünschte Antworten – etwa zur Bewertung von Bezugsquellen – begünstigt haben könnte.

Die Einteilung in „legal“ und „illegal“ ist mit Unsicherheiten verbunden, etwa beim tatsächlichen Einhalten der Vorgaben zum Eigenanbau. Zudem war die Frage zum höchsten Berufsabschluss nicht eindeutig differenziert. Freitextangaben (Meister, Techniker) wurden der Berufsausbildung zugeordnet.

Aufgrund der Vielzahl durchgeführter statistischer Tests ist die Wahrscheinlichkeit falsch-positiver Befunde erhöht. Da die Analysen explorativ sind, wurden keine Korrekturen für multiples Testen vorgenommen und die *p*-Werte sind entsprechend vorsichtig zu interpretieren.

## Fazit

Insgesamt zeigt sich, dass eine große Mehrheit der regelmäßig Konsumierenden auf legale Bezugswege umgestiegen ist. Die Ergebnisse deuten darauf hin, dass für bestimmte Gruppen Barrieren im Zugang zum legalen Markt bestehen, darunter Jugendliche, Frauen und die urbane Bevölkerung. Für diese Gruppen könnten gezielte Maßnahmen zum Abbau von Barrieren einen Beitrag zur Zurückdrängung illegaler Bezugswege leisten.

## Supplementary Information

Onlinematerial: Fragebogen Cannabiskonsum

## Data Availability

Die während der vorliegenden Studie erzeugten und/oder analysierten Datensätze sind auf begründete Anfrage bei der Korrespondenzperson erhältlich.
