# Supplementary material for: Effects of the cannabis act on supply channels, attitudes, and perceptions of users
Source: Bundesgesundheitsblatt Gesundheitsforschung Gesundheitsschutz. 2026 Jun 11;69(7):794–802. [Article in German] doi: 10.1007/s00103-026-04258-y (PMC13323109; doi:10.1007/s00103-026-04258-y)
Supplement: Supplementary file 1 — Onlinematerial: Fragebogen Cannabiskonsum [file 103_2026_4258_MOESM1_ESM.pdf]

Fragebogen zur Studie „Veränderungen für Konsumierende von Cannabis durch das Cannabisgesetz (KonCanG)"

Q1	**Veränderungen für Konsumierende von Cannabis durch das Cannabisgesetz (KonCanG)**<br>Mit Inkrafttreten des Cannabisgesetzes zum 1. April 2024 hat sich für Konsumierende einiges verändert. Mit dieser Befragung möchten wir diese Veränderungen erfassen.<br>Diese Umfrage richtet sich daher an Personen ab 14 Jahren, die seit dem 01.04.2024 (Inkrafttreten des Cannabisgesetzes) Cannabis konsumiert haben. Ausgeschlossen sind Personen die keine THC-haltigen Produkte, sondern nur (teil-)synthetische Cannabinoide konsumieren.<br>Die Teilnahme an der Umfrage ist selbstverständlich freiwillig und anonym. Es werden auch keine IP-Adressen o. ä. gesammelt. Die erhobenen Daten lassen keinen Rückschluss auf Ihre Person zu. Die Ergebnisse der Umfrage werden anschließend der Fachöffentlichkeit, z. B. im Rahmen von wissenschaftlichen Publikationen, zur Verfügung gestellt.<br>Die Befragung dauert ca. 10 Minuten. Bitte lesen Sie sich die Fragen sorgfältig durch.<br>Die Befragung wird von Prof. Dr. Bernd Werse (Frankfurt University of Applied Sciences), Prof.in Dr.in Anke Stallwitz (Evangelische Hochschule Freiburg) und Larissa Steimle (Frankfurt University of Applied Sciences) durchgeführt und von der Frankfurt University of Applied Sciences finanziert.<br>Fragen zur Erhebung richten Sie bitte an.
Q2	Haben Sie seit dem 01.04.2024 mindestens einmal Cannabis (Marihuana, Gras, Haschisch, Extrakte etc.) konsumiert?<br>Dabei geht es nicht um Cannabisprodukte mit geringem THC-Gehalt wie etwa "CBD-Gras" oder (teil-)synthetische Cannabinoide.<br>1. Ja<br>2. Nein<br>*Wenn Nein ist ausgewählt gehe zu Ende der Umfrage*
Q3	Haben Sie seit dem 01.04.2024 mindestens eine der folgenden Produktarten konsumiert? (Mehrfachnennungen möglich)<br>1. HHC oder ähnliche Derivate (z.B. HHCP, 10-OH-HHC, 10-OH-THC) – Blüten, Hasch, Reinsubstanz oder Vape-Pens<br>2. Andere synthetische Cannabinoide/Cannabinoidmimetika - als Räuchermischungen oder Reinsubstanz<br>3. Keine der Produktarten
Q4	Haben Sie **vor** dem 01.04.2024 mindestens einmal Cannabis konsumiert?

	Dabei geht es <u>nicht</u> um Cannabisprodukte mit geringem THC-Gehalt wie etwa "CBD-Gras" oder (teil-)synthetische Cannabinoide.<br>1. Ja<br>2. Nein
Q5	Haben Sie **<u>vor</u>** <u>dem 01.04.2024</u> mindestens einmaleine der folgenden Produktarten konsumiert? (Mehrfachnennungen möglich)<br>1. HHC oder ähnliche Derivate (z.B. HHCP, 10-OH-HHC, 10-OH-THC) – Blüten, Hasch, Reinsubstanz oder Vape-Pens<br>2. Andere synthetische Cannabinoide/Cannabinoidmimetika - als Räuchermischungen oder Reinsubstanz<br>3. Keine der Produktarten
Q6	Wie alt sind Sie?<br>1. Jünger als 14 Jahre<br>2. Älter als 14 Jahre, jünger als 18 Jahre, nämlich: ___<br>3. Älter als 18 Jahre, nämlich: ___<br>*Wenn Jünger als 14 Jahre ist ausgewählt gehe zu Ende der Umfrage*
Q7	Wann haben Sie <u>zum letzten Mal</u> Cannabis konsumiert?<br>Dabei geht es <u>nicht</u> um Cannabisprodukte mit geringem THC-Gehalt wie etwa "CBD-Gras" oder (teil-)synthetische Cannabinoide.<br>1. In den letzten 24 Stunden<br>2. In den letzten 30 Tagen, aber nicht in den letzten 24 Stunden<br>3. In den letzten 12 Monaten, aber nicht in den letzten 30 Tagen
Q8	Wie häufig konsumieren Sie Cannabis?<br>Dabei geht es <u>nicht</u> um Cannabisprodukte mit geringem THC-Gehalt wie etwa "CBD-Gras" oder (teil-)synthetische Cannabinoide.<br>1. Täglich<br>2. Mehrmals pro Woche<br>3. Ungefähr einmal pro Woche<br>4. Nicht wöchentlich, aber mindestens einmal pro Monat<br>5. Nicht monatlich, aber mindestens einmal pro Jahr
Q9	Wie viel Cannabis konsumieren Sie (ungefähr, durchschnittlich) pro Tag?<br>Bitte mit einer Nachkommastelle angeben.

	___ Gramm<br>*Frage anzeigen nur, wenn diese Bedingung erfüllt ist: Q8 Auswahlmöglichkeit „1. Täglich" ist ausgewählt*
Q10	In welcher Form konsumieren Sie Cannabis? (Mehrfachnennungen möglich)<br>1. Joint mit Tabak<br>2. Joint ohne Tabak<br>3. Vaporizer<br>4. Edibles oder Getränke (z. B. Haschkekse, Kakao o. ä.)<br>5. Dabbing (Verdampfen und Inhalieren von Konzentraten)<br>6. E-Zigaretten/Vapes<br>7. Pfeife<br>8. Wasserpfeife (Bong)<br>9. Sonstiges: ___
Q11	In welcher Form konsumieren Sie Cannabis <u>hauptsächlich</u>?<br>1. Joint mit Tabak<br>2. Joint ohne Tabak<br>3. Vaporizer<br>4. Edibles oder Getränke (z. B. Haschkekse, Kakao o. ä.)<br>5. Dabbing (Verdampfen und Inhalieren von Konzentraten)<br>6. E-Zigaretten/Vapes<br>7. Pfeife<br>8. Wasserpfeife (Bong)<br>9. Sonstiges: ___
Q12	Wie sind Sie das erste Mal mit Cannabis in Kontakt gekommen?<br>1. Freunde/Freundinnen<br>2. Bekannte/Mitschüler/innen<br>3. Eltern<br>4. Geschwister/Cousins/Cousinen o. ä.<br>5. Dealer/Dealerinnen<br>6. Weiß ich nicht<br>7. Sonstige: ___

	Frage anzeigen nur, wenn diese Bedingung erfüllt ist: Q6 Auswahlmöglichkeit „2. Älter als 14, jünger als 18 Jahre, nämlich“ ist ausgewählt
Q13	Konsumiert mindestens eines Ihrer Elternteile/Erziehungsberechtigte (zumindest gelegentlich) Cannabis?<br>1. Ja<br>2. Nein<br>3. Weiß ich nicht<br>*Frage anzeigen nur, wenn diese Bedingung erfüllt ist: Q6 Auswahlmöglichkeit „2. Älter als 14, jünger als 18 Jahre, nämlich“ ist ausgewählt*
Q14	Haben Sie über Ihre Eltern Zugang zu Cannabisprodukten?<br>1. Ja, sie erlauben mir, ihre Vorräte zu verwenden<br>2. Ja, ich kann mich an ihren Vorräten bedienen, sie haben es mir aber nicht ausdrücklich erlaubt<br>3. Nein<br>*Frage anzeigen nur, wenn diese Bedingung erfüllt ist: Q6 Auswahlmöglichkeit „2. Älter als 14, jünger als 18 Jahre, nämlich“ ist ausgewählt*<br>*Frage anzeigen nur, wenn diese Bedingung erfüllt ist: Q13 Auswahlmöglichkeit „1. Ja“ ist ausgewählt*
Q15	Haben Sie schon einmal mit Ihren Eltern zusammen konsumiert?<br>1. Ja, einmal<br>2. Ja, schon mehrmals<br>3. Nein<br>*Frage anzeigen nur, wenn diese Bedingung erfüllt ist: Q6 Auswahlmöglichkeit „2. Älter als 14, jünger als 18 Jahre, nämlich“ ist ausgewählt*<br>*Frage anzeigen nur, wenn diese Bedingung erfüllt ist: Q13 Auswahlmöglichkeit „1. Ja“ ist ausgewählt*
Q16	Aus welchen Quellen haben Sie vor dem 01.04.2024 Ihr Cannabis bezogen? (Mehrfachnennungen möglich)<br>1. Ich habe zu dieser Zeit nicht konsumiert<br>2. Freunde/Bekannte<br>3. Eltern oder andere ältere Verwandte<br>4. Dealer/innen, die bekannt/vertraut sind<br>5. Dealer in der Öffentlichkeit<br>6. Darknet/illegale Online-Shops<br>7. Social Media (Telegram etc.)<br>8. Eigenanbau (von Ihnen selbst betrieben)<br>9. Ausland

	10. Weiß ich nicht<br>11. Sonstiges: ___<br>*Frage anzeigen nur, wenn diese Bedingung erfüllt ist: Q6 Auswahlmöglichkeit „2. Älter als 14, jünger als 18 Jahre, nämlich“ ist ausgewählt*
Q17	Was war die Hauptquelle, aus der Sie vor dem 01.04.2024 Ihr Cannabis bezogen?<br>1. Ich habe zu dieser Zeit nicht konsumiert<br>2. Freunde/Bekannte<br>3. Eltern oder andere ältere Verwandte<br>4. Dealer/innen, die bekannt/vertraut sind<br>5. Dealer in der Öffentlichkeit<br>6. Darknet/illegale Online-Shops<br>7. Social Media (Telegram etc.)<br>8. Eigenanbau (von Ihnen selbst betrieben)<br>9. Ausland<br>10. Weiß ich nicht<br>11. Sonstiges: ___<br>*Frage anzeigen nur, wenn diese Bedingung erfüllt ist: Q6 Auswahlmöglichkeit „2. Älter als 14, jünger als 18 Jahre, nämlich“ ist ausgewählt*
Q18	Aus welcher Quelle haben Sie in den letzten sechs Monaten Ihr Cannabis bezogen? (Mehrfachnennungen möglich)<br>Hier geht es darum, zu erfassen, ob sich Ihre Bezugsquelle seit dem Cannabisgesetz verändert hat. Da uns bewusst ist, dass es eine gewisse Übergangsphase gegeben hat, interessieren uns vorrangig die letzten sechs Monate.<br>1. Freunde/Bekannte<br>2. Eltern oder andere ältere Verwandte<br>3. Dealer/innen, die bekannt/vertraut sind<br>4. Dealer in der Öffentlichkeit<br>5. Darknet/illegale Online-Shops<br>6. Social Media (Telegram etc.)<br>7. Eigenanbau (von Ihnen selbst betrieben)<br>8. Ausland<br>9. Weiß ich nicht<br>10. Sonstiges: ___<br>*Frage anzeigen nur, wenn diese Bedingung erfüllt ist: Q6 Auswahlmöglichkeit „2. Älter als 14, jünger als 18 Jahre, nämlich“ ist ausgewählt*

Q19	Was ist aktuell die Hauptquelle, aus der Sie Cannabis beziehen?<br>1. Freunde/Bekannte<br>2. Eltern oder andere ältere Verwandte<br>3. Dealer/innen, die bekannt/vertraut sind<br>4. Dealer in der Öffentlichkeit<br>5. Darknet/illegale Online-Shops<br>6. Social Media (Telegram etc.)<br>7. Eigenanbau (von Ihnen selbst betrieben)<br>8. Ausland<br>9. Weiß ich nicht<br>10. Sonstiges: ___<br>*Frage anzeigen nur, wenn diese Bedingung erfüllt ist: Q6 Auswahlmöglichkeit „2. Älter als 14, jünger als 18 Jahre, nämlich“ ist ausgewählt*
Q20	Wissen Sie, woher ihre Freunde/Bekannte/Eltern/andere ältere Verwandte das Cannabis haben? (Mehrfachnennungen möglich)<br>1. Selbst angebaut<br>2. Aus Anbauvereinigung<br>3. Apotheke<br>4. Illegale Quelle<br>5. Weiß ich nicht<br>*Frage anzeigen nur, wenn diese Bedingung erfüllt ist: Q6 Auswahlmöglichkeit „2. Älter als 14, jünger als 18 Jahre, nämlich“ ist ausgewählt*<br>*Frage anzeigen nur, wenn diese Bedingung erfüllt ist: Q19 Auswahlmöglichkeit „2. Eltern oder andere ältere Verwandte“ ist ausgewählt*<br>*Frage anzeigen nur, wenn diese Bedingung erfüllt ist: Q19 Auswahlmöglichkeit „1. Freunde/Bekannte“ ist ausgewählt*
Q21	Aus welchen Quellen haben Sie vor dem 01.04.2024 Ihr Cannabis bezogen? (Mehrfachnennungen möglich)<br>1. Ich habe zu dieser Zeit nicht konsumiert<br>2. Freunde/Bekannte (Cannabis, das diese selbst angebaut haben)<br>3. Freunde/Bekannte (nicht selbst angebautes Cannabis)<br>4. Dealer/innen, die bekannt/vertraut sind<br>5. Dealer in der Öffentlichkeit<br>6. Darknet/illegale Online-Shops<br>7. Social Media (Telegram etc.)<br>8. Eigenanbau

	9. Apotheke<br>10. Ausland<br>11. Sonstiges: ___<br><br>*Frage anzeigen nur, wenn diese Bedingung erfüllt ist: Q6 Auswahlmöglichkeit „3. Älter als 18 Jahre, nämlich“ ist ausgewählt*
Q22	Was war die Hauptquelle, aus der Sie vor dem 01.04.2024 Ihr Cannabis bezogen?<br><br>1. Ich habe zu dieser Zeit nicht konsumiert<br>2. Freunde/Bekannte (Cannabis, das diese selbst angebaut haben)<br>3. Freunde/Bekannte (nicht selbst angebautes Cannabis)<br>4. Dealer/innen, die bekannt/vertraut sind<br>5. Dealer in der Öffentlichkeit<br>6. Darknet/illegale Online-Shops<br>7. Social Media (Telegram etc.)<br>8. Eigenanbau<br>9. Apotheke<br>10. Ausland<br>11. Sonstiges: ___<br><br>*Frage anzeigen nur, wenn diese Bedingung erfüllt ist: Q6 Auswahlmöglichkeit „3. Älter als 18 Jahre, nämlich“ ist ausgewählt*
Q23	Aus welcher Quelle haben Sie in den letzten sechs Monaten Ihr Cannabis bezogen? (Mehrfachnennungen möglich)<br><br>Hier geht es darum, zu erfassen, ob sich Ihre Bezugsquelle seit dem Cannabisgesetz verändert hat. Da uns bewusst ist, dass es eine gewisse Übergangsphase gegeben hat, interessieren uns vorrangig die letzten sechs Monate.<br><br>1. Freunde/Bekannte (Cannabis, das diese selbst angebaut haben)<br>2. Freunde/Bekannte (nicht selbst angebautes Cannabis)<br>3. Dealer/innen, die bekannt/vertraut sind<br>4. Dealer in der Öffentlichkeit<br>5. Darknet/illegale Online-Shops<br>6. Social Media (Telegram etc.)<br>7. Eigenanbau<br>8. Apotheke<br>9. Ausland<br>10. Anbauvereinigung<br>11. Sonstiges: ___

	Frage anzeigen nur, wenn diese Bedingung erfüllt ist: Q6 Auswahlmöglichkeit „3. Älter als 18 Jahre, nämlich“ ist ausgewählt
Q24	Wo bauen Sie Ihr Cannabis an? (Mehrfachnennungen möglich)<br>1. Indoor (z. B. in der eigenen Wohnung, mit künstlicher Beleuchtung)<br>2. Outdoor (z. B. im Garten, auf dem Balkon oder im Gewächshaus)<br>*Frage anzeigen nur, wenn diese Bedingung erfüllt ist: Q6 Auswahlmöglichkeit „3. Älter als 18 Jahre, nämlich“ ist ausgewählt*<br>*Frage anzeigen nur, wenn diese Bedingung erfüllt ist: Q23 Auswahlmöglichkeit „7. Eigenanbau“ ist ausgewählt*
Q25	Wie viele Ernten haben Sie seit dem 01.04.2024 eingefahren?<br>1. Eine<br>2. Zwei<br>3. Drei<br>4. Vier oder mehr<br>5. Kann ich nicht genau sagen, habe bei einzelnen Grows schrittweise geerntet<br>*Frage anzeigen nur, wenn diese Bedingung erfüllt ist: Q6 Auswahlmöglichkeit „3. Älter als 18 Jahre, nämlich“ ist ausgewählt*<br>*Frage anzeigen nur, wenn diese Bedingung erfüllt ist: Q23 Auswahlmöglichkeit „7. Eigenanbau“ ist ausgewählt*
Q26	Haben Sie für Ihren Outdoor-Anbau Sicherheitsmaßnahmen unternommen, um den Zugriff für fremde Personen einzuschränken?<br>1. Nein, keine Maßnahmen<br>2. Bereich ist ohnehin unzugänglich (z. B. Balkon oder gut gesicherter Garten)<br>3. Anbau an nicht von außen einsehbarer Stelle<br>4. Anbau schwer erkennbar zwischen anderen Pflanzen<br>5. Zusätzliche Umzäunung oder Schlösser angebracht<br>6. Videoüberwachung<br>7. Sonstige: ___<br>*Frage anzeigen nur, wenn diese Bedingung erfüllt ist: Q6 Auswahlmöglichkeit „3. Älter als 18 Jahre, nämlich“ ist ausgewählt*<br>*Frage anzeigen nur, wenn diese Bedingung erfüllt ist: Q24 Auswahlmöglichkeit „2. Outdoor“ ist ausgewählt*
Q27	Haben Sie Sicherheitsmaßnahmen unternommen, um den Zugriff für Kinder einzuschränken?<br>1. Nicht relevant für mich, Kinder haben sowieso keinen Zugang<br>2. Nein, keine Maßnahmen<br>3. Grow-Bereich ist abschließbar

	4. Grow-Bereich ist versteckt/nicht zugänglich für Kinder<br>5. Sonstige: ___<br>*Frage anzeigen nur, wenn diese Bedingung erfüllt ist: Q6 Auswahlmöglichkeit „3. Älter als 18 Jahre, nämlich“ ist ausgewählt*<br>*Frage anzeigen nur, wenn diese Bedingung erfüllt ist: Q23 Auswahlmöglichkeit „7. Eigenanbau“ ist ausgewählt*
Q28	Haben Sie ein Rezept mit Kostenübernahme der Krankenkasse für Ihren medizinischen Cannabisgebrauch?<br>1. Ja<br>2. Nein<br>*Frage anzeigen nur, wenn diese Bedingung erfüllt ist: Q6 Auswahlmöglichkeit „3. Älter als 18 Jahre, nämlich“ ist ausgewählt*<br>*Frage anzeigen nur, wenn diese Bedingung erfüllt ist: Q23 Auswahlmöglichkeit „8. Apotheke“ ist ausgewählt*
Q29	Was ist aktuell die Hauptquelle, aus der Sie Cannabis beziehen?<br>1. Freunde/Bekannte (Cannabis, das diese selbst angebaut haben)<br>2. Freunde/Bekannte (nicht selbst angebautes Cannabis)<br>3. Dealer/innen, die bekannt/vertraut sind<br>4. Dealer in der Öffentlichkeit<br>5. Darknet/illegale Online-Shops<br>6. Social Media (Telegram etc.)<br>7. Eigenanbau<br>8. Apotheke<br>9. Ausland<br>10. Anbauvereinigung<br>11. Sonstiges: ___<br>*Frage anzeigen nur, wenn diese Bedingung erfüllt ist: Q6 Auswahlmöglichkeit „3. Älter als 18 Jahre, nämlich“ ist ausgewählt*
Q30	Sind Sie Mitglied in einer Anbauvereinigung?<br>1. Ja<br>2. Nein<br>*Frage anzeigen nur, wenn diese Bedingung erfüllt ist: Q6 Auswahlmöglichkeit „3. Älter als 18 Jahre, nämlich“ ist ausgewählt*
Q31	An welchen Orten konsumieren Sie? (Mehrfachnennungen möglich)<br>1. Auf dem eigenen Grundstück (in der Wohnung, im eigenen Garten etc.)

	2. Im öffentlichen Raum<br>3. In der Gastronomie<br>4. Bei Freundinnen und Freunden<br>5. Sonstige: ___
Q32	Aus welchen Gründen konsumieren Sie Cannabis? (Mehrfachnennungen möglich)<br>1. Weil meine Freundinnen und Freunde auch konsumieren<br>2. Weil ich das Gefühl mag<br>3. Um meine eigenen Sorgen vergessen zu können<br>4. Ich mag es, gemeinsam mit anderen zu konsumieren<br>5. Um mich zu entspannen<br>6. Um meine Wahrnehmung zu verändern<br>7. Aus medizinischen Gründen<br>8. Aus Gewohnheit<br>9. Sonstiges: ___
Q33	Was hat sich seit der Einführung des Cannabisgesetzes für Sie verändert?

	Stimme überhaupt nicht zu	Stimme nicht zu	Stimme weder zu noch lehne ab	Stimme zu	Stimme voll und ganz zu
Ich fühle mich weniger beobachtet beim Konsum					
Ich fühle mich akzeptierter als konsumierende Person					
Ich habe keine Angst mehr vor Strafverfolgung, wenn ich Cannabis dabei habe					

	Seit der Änderung der Fahrerlaubnisverordnung und der Erhöhung des THC-Grenzwertes habe ich keine Sorgen mehr, wenn ich motorisiert im Straßenverkehr unterwegs bin (Falls Sie sich nicht motorisiert im Straßenverkehr bewegen, bitte leer lassen)					
	Ich habe weniger Hemmungen, mir Hilfe zu suchen, falls mir mein Konsum Probleme bereiten würde					
Q34	Zum Abschluss benötigen wir noch ein paar soziodemographische Daten von Ihnen.					
Q35	Welchem Geschlecht fühlen Sie sich zugehörig?<br>1. Weiblich<br>2. Männlich<br>3. Divers: ___					
Q36	Welchen höchsten Bildungsabschluss haben Sie?<br>Wenn Sie einen Abschluss aus einem anderen Land haben, wählen Sie bitte dessen Äquivalent aus dem deutschen Schulsystem.<br>1. Aktuell in der Schule<br>2. Kein Schulabschluss<br>3. Hauptschulabschluss (bzw. POS 8./9. Klasse)<br>4. Realschulabschluss (Mittlere Reife bzw. POS 10. Klasse)<br>5. Abschluss einer Fachoberschule<br>6. Abitur oder Fachhochschulreife (bzw. EOS)<br>7. Universitäts- oder Hochschulabschluss (Diplom, Bachelor, Master, Doktor, Staatsexamen etc.)<br>8. Abgeschlossene Berufsausbildung<br>9. Einen anderen Abschluss: ___					
Q37	Wie hoch ist Ihr monatliches Netto-Einkommen Ihres Haushalts insgesamt?<br>Gemeint ist dabei die Summe, die sich aus Lohn, Gehalt, Einkommen aus selbständiger Tätigkeit, Rente oder Pension ergibt. Rechnen Sie bitte auch die Einkünfte aus öffentlichen Beihilfen, Einkommen aus Vermietung, Verpachtung, Wohngeld, Kindergeld und sonstige Einkünfte hinzu und ziehen Sie dann Steuern und Sozialversicherungsbeiträge ab.<br>1. 0€ - 1.500€<br>2. 1.501€ - 3.000€					

	3. 3.001€ - 5.000€<br>4. 5.001€ oder mehr
Q38	Welche Staatsbürgerschaft haben Sie?<br>1. Deutsch<br>2. Andere: ___<br>3. Doppelte, nämlich: ___
Q39	Wo wohnen Sie?<br>1. In einer Stadt/einem Dorf mit bis zu 10.000 Einwohnern<br>2. In einer Stadt von 10.000 bis 100.00 Einwohnern<br>3. In einer Großstadt mit min. 100.000 Einwohnern
Q40	In welchem Bundesland leben Sie?<br>1. Baden-Württemberg<br>2. Bayern<br>3. Berlin<br>4. Brandenburg<br>5. Bremen<br>6. Hamburg<br>7. Hessen<br>8. Mecklenburg-Vorpommern<br>9. Niedersachsen<br>10. Nordrhein-Westfalen<br>11. Rheinland-Pfalz<br>12. Saarland<br>13. Sachsen<br>14. Sachsen-Anhalt<br>15. Schleswig-Holstein<br>16. Thüringen<br>17. Ausland: ___
Q41	Leben Minderjährige in Ihrem Haushalt?

	1. Ja<br>2. Nein<br>3. Keine Angabe<br><br>*Frage anzeigen nur, wenn diese Bedingung erfüllt ist: Q6 Auswahlmöglichkeit „3. Älter als 18 Jahre, nämlich“ ist ausgewählt*
Q42	Haben Sie weitere Anmerkungen zum Thema?<br><br>___
